# Reversing Adhesion: A Triggered Release Self‐Reporting Adhesive

**DOI:** 10.1002/advs.201500361

**Published:** 2016-02-02

**Authors:** Alexander M. Schenzel, Christopher Klein, Kai Rist, Norbert Moszner, Christopher Barner‐Kowollik

**Affiliations:** ^1^ Preparative Macromolecular ChemistryInstitut für Technische Chemie und PolymerchemieKarlsruhe Institute of Technology (KIT)Engesserstr. 1876128KarlsruheGermany; ^2^ Institut für Biologische GrenzflächenKarlsruhe Institute of Technology (KIT)Hermann‐von‐Helmholtz‐Platz 176344Eggenstein‐LeopoldshafenGermany; ^3^ Polymeric MaterialsInstitut für Technische Chemie und PolymerchemieKarlsruhe Institute of Technology (KIT)Engesserstr. 1876128KarlsruheGermany; ^4^ Ivoclar Vivadent AGBendererstr. 29494SchaanLiechtenstein

**Keywords:** degradable networks, hetero Diels–Alder chemistry, polymer chemistry, self‐reporting materials

## Abstract

Here, the development of an adhesive is reported – generated via free radical polymerization – which can be degraded upon thermal impact within minutes. The degradation is based on a stimuli responsive moiety (SRM) that is incorporated into the network. The selected SRM is a hetero Diels‐Alder (HDA) moiety that features three key properties. First, the adhesive can be degraded at relatively low temperatures (≈80 °C), second the degradation occurs very rapidly (less than 3 min), and third, the degradation of the network can readily be analyzed and quantified due to its self‐reporting nature. The new reversible self‐reporting adhesion system is characterized in detail starting from molecular studies of the retro HDA reaction. Moreover, the mechanical properties of the network, as well as the adhesion forces, are investigated in detail and compared to common methacrylate‐based systems, demonstrating a significant decrease in mechanic stability at elevated temperatures. The current study thus represents a significant advance of the current state of the art for debonding on demand adhesives, making the system interesting for several fields of application including dental adhesives.

Adhesives are essential in our everyday life due to their wide scope of application. From a chemical point of view, adhesives prepared via free‐radical polymerization usually contain monomers that form a polymeric network upon curing. Covalent polymeric networks are generally not alterable once the network is formed as macromolecular chains are irreversibly crosslinked, leading to nonrevocable and permanent adhesion. Here, we report the development of an adhesive‐generated via free radical polymerization, which can be degraded upon thermal impact within minutes. The degradation is based on a stimuli responsive moiety (SRM) that is incorporated into the network. The selected SRM features three key properties. First, the adhesive can be degraded at relatively low temperatures (≈80 °C), second the degradation occurs very rapidly (less than 3 min), and third, the degradation of the network can readily be analyzed and quantified due to its self‐reporting nature, representing a significant advance over the current state of the art. The present study quantifies the performance of the new reversible self‐reporting adhesion system from the molecular level to application tests.

Numerous products of our daily life, such as mobile phones, cars, and floorings would not be producible as we know them today, if adhesives did not exist. By definition, an adhesive permanently binds two materials when applied to their respective surfaces.^1^ For most applications, permanent adhesion is needed and a separation in the future is not desired or even unwanted. However, for some tasks an adhesive which shows excellent bonding performance yet can be removed easily when required is of high interest. For example, today, high forces and time consuming grinding are necessary to remove dental ceramic crowns or orthodontic brackets completely, increasing the risk of damage for the teeth. A readily removable adhesive is highly desired for the bonding of such materials, as it would ease the removal for the dentist and reduce the force applied to the teeth.

However, such a debonding on demand (DoD) adhesive should not degrade spontaneously. Therefore, degradation has to be induced by a well‐controllable trigger system. In general, triggers such as light,^2^ heat,^3^ pH changes,^4^ or an acid/base reaction^5^ can be employed to alter polymeric structures by breaking covalent bonds. In order to design a DoD adhesive, triggering the SRM must lead to debonding of the polymeric network and corresponding debonding of the thereon based adhesive. When vinylic monomers are employed, the SRM can be incorporated into a crosslinker, which is typically a di‐ or polyvinylic monomer.

In recent years several attempts have been made to prepare degradable networks.^6^ However, degradable systems known today require relatively high temperatures^7^ and/or many hours^8^ or days[[qv: 5a]] to cleave, which in most cases limits their applicability drastically.

Inspired by the goal to overcome these limitations, we present a DoD adhesive that can be degraded in minutes when heated to 80 °C (see **Figure** **1**).

**Figure 1:**
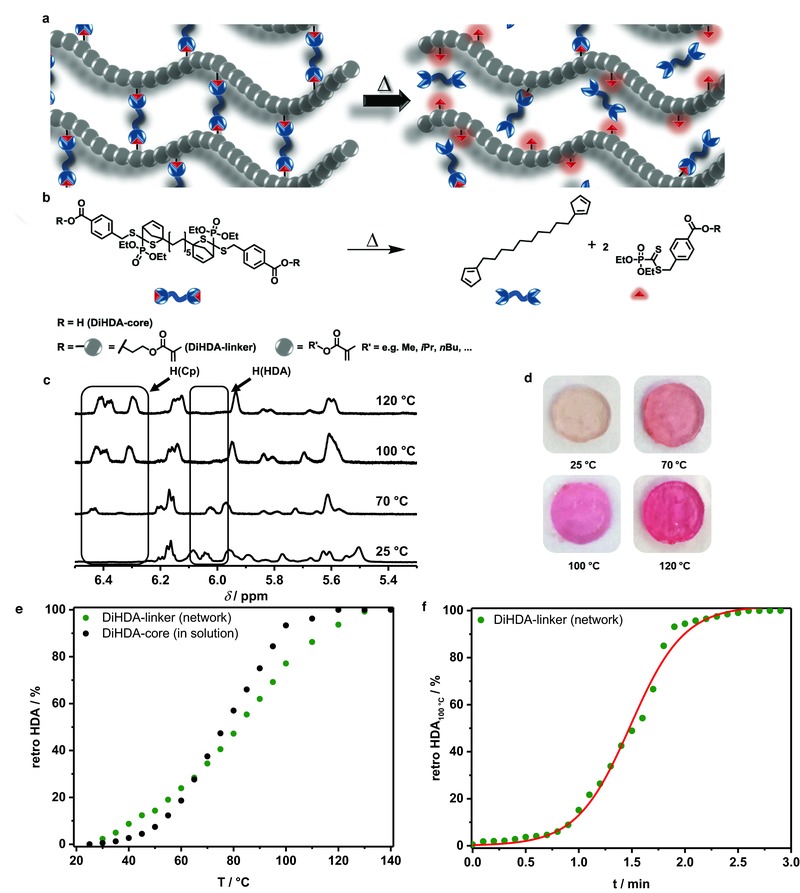
A self‐reporting degradable polymeric network including spectroscopic and visual analysis. a) Schematic display of the degradation of the network upon heating. b) Display of the retro HDA reaction that occurs upon heating and the defined structures of the DiHDA‐core and ‐linker (the DiHDA‐core was used for temperature dependent studies in solution in order to avoid any self‐polymerization). The self‐reporting nature stems from the generated dithioester 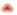 formed upon heating. c) HT‐NMR spectroscopic analysis of the DiHDA‐core in toluene‐d_8_ at 25, 70, 100, and 120 °C. d) Evidence of the color change upon heating at 25 °C (0%*), 70 °C (34%*), 100 °C (93%*), and 120 °C (100%*). e) UV–Vis spectroscopic analysis of the DiHDA‐core in solution and a cured network of the DiHDA‐linker (99.8% DiHDA‐linker + 0.2% Ivocerin) between 25 and 140 °C.** f) Kinetic investigation of the retro HDA reaction of a cured network of the DiHDA‐linker (99.8% DiHDA‐linker + 0.2% Ivocerin) at 100 °C. *Amount of retro HDA product. **The intensity of the maxima at 330 nm (DiHDA‐core) and 535 nm (DiHDA‐linker) is observed at a given temperature and correlated to the extent of the retro HDA reaction. For the DiHDA‐core, the absorption at 330 nm was chosen as the absorption at 535 nm was weak, due to poor solubility.

The new adhesive is based on a polymer network formed via free radical polymerization of a dimethacrylate crosslinker including two thermally sensitive hetero Diels–Alder (HDA) moieties (DiHDA‐linker). A methacrylate species was chosen due to its low toxicity, making the system also interesting for biomedical applications. The HDA groups, which are incorporated in the linker can be cleaved due to the retro HDA reaction, which occurs upon heating (refer to Figure 1b). The cleavage leads to the desired degradation and debonding of the network and the concomitant formation of highly colored (red) dithioester species (refer to Figure 1d), indicating the release of the adhesive in a simple visual inspection system. In order to increase the cleavage efficiency within the network, two HDA groups are incorporated into one linker, since a cleavage of only one HDA functionality would already lead to a cleavage of the crosslinks between the polymeric chains. The synthetic protocol for the dimethacrylate crosslinker (DiHDA‐linker) and the prepared polymeric networks as well as the detailed reaction procedures can be found in the Supporting Information.

Prior to the adhesion and network release studies, the SRM has to be characterized in detail in order to confirm and quantify the debonding performance of the molecular system. Therefore, the HDA‐moiety was analyzed with high temperature (HT)‐NMR and UV/Vis spectroscopy. In order to prevent an undesired polymerization at elevated temperatures, a precursor (DiHDA‐core), instead of the vinyl functional linker itself was investigated (refer to Figure 1b). Since the substitution close to the HDA‐moiety is identical in both molecules, the DiHDA‐linker displays the same behavior as the DiHDA‐core. HT‐NMR spectroscopic measurements of the DiHDA‐core reveal that the HDA equilibrium is completely shifted to the side of the HDA product at temperatures below 25 °C. When the HDA moiety is heated, the concentration of retro HDA products increases steadily until complete conversion is reached at 120 °C (Figure 1c).

Due to the absorption of the formed C=S double bond in the visible light range (535 nm), the retro HDA reaction induces a color change, giving the adhesive release system its self‐reporting nature. The change in color upon heating can readily be seen with the naked eye, making the system ideally suited for a range of applications, as it provides the user with a visible inspection system for the degree of debonding (refer to Figure 1d).

UV/Vis spectroscopic measurements can quantify the visibly observable color change. For networks based on a DiHDA‐linker, the measurement can be carried out when the network is directly formed in a UV/Vis cuvette. The amount of retro HDA product can be deduced directly at any given temperature as the start of the retro reaction (no absorption) and complete conversion (maximum absorption) can be determined during the measurement (refer to Figure 1e and the Supporting Information). The start and end temperature of the retro HDA reaction assigned in the UV/Vis spectroscopic measurement correlates with the values determined in the HT‐NMR analysis. When compared to the core in solution, the linker network shows very similar behavior. However, a higher degree of debonding of the DiHDA‐linker network can be detected at temperatures below 65 °C. Such an observation is consistent with the fact that a detachment in a network leads to a higher entropy release compared to a detachment from a molecule in solution. A more detailed study of the entropic effect operational in cleaving macromolecular systems was carried out by our research group in a series of previous studies.^9^

In addition to the spectroscopic studies, the degradation as a function of time was determined as well. The time required for the degradation of the adhesive is a crucial point if the adhesive release system is to be applicable in a practical context. However, most of the degradable networks known today require several hours or days to debond completely.[[qv: 5a,c]] As the retro HDA reaction is known to be quite rapid in the current system, this limitation should be overcome with the invented adhesive. Therefore, the kinetics of the retro HDA reaction in the polymeric network were investigated (refer to Figure 1f). At 100 °C, the equilibrium is reached in less than 3 min, demonstrating that the debonding takes place very rapidly, making the HDA system highly interesting for real life applications.

In order to evidence that the fast cleavage of the employed SRM can lead to an efficient degradation of a polymeric network, its mechanical properties upon application of the degradation trigger were carefully studied. If the degradation is successful, a clear decrease in mechanical stability should be detectable, with the same change not evident in a reference systems containing a noncleavable linker.

Thus, rheological measurements of the prepared materials were carried out (for more details, refer to the Supporting Information). When compared to a typical, nondegradable polymer network based on urethane dimethacrylate (UDMA), the differences in the mechanical properties are clearly visible (see **Figure** **2**a). Here, the storage modulus G′ associated with the elastic contribution of the stress response of the sample and therefore its stability is plotted against the temperature. As expected for the nondegradable reference network, the storage modulus of the UDMA network decreases only slightly when the temperature increases. For the DiHDA‐linker based network, a drastic loss in G′ by over two orders of magnitude (from 8.5 × 10^8^ Pa at 25 °C to 5.5 × 10^6^ Pa at 120 °C) and therefore in the stability of the network is observed upon heating, evidencing that substantial degradation of the adhesive takes place when the sample is heated.

**Figure 2:**
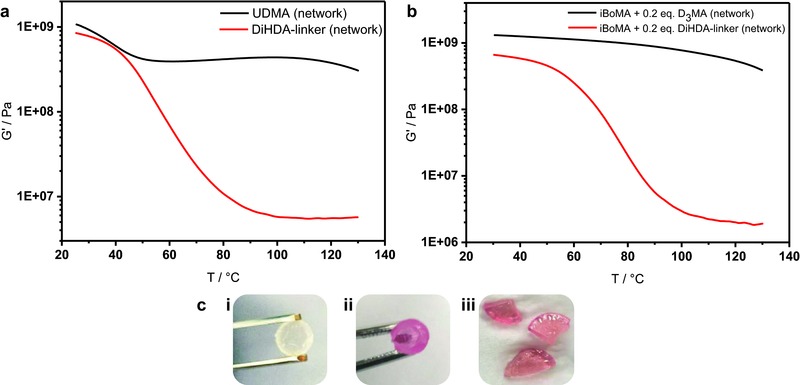
Analysis of the mechanical network properties. a) Comparison of the storage modulus (G′) of a degradable network (99.8% DiHDA‐linker + 0.2% Ivocerin) and a typical nondegradable polymeric network (99.8% UDMA + 0.2% Ivocerin) for the temperature range of 25–130 °C. b) Comparison of G′ of a degradable copolymer network (*i*BoMA + 0.2 equation DiHDA‐linker) and a typical nondegradable copolymer network (*i*BoMA + 0.2 equation D_3_MA*) for the temperature range of 30–130 °C. c) Display of a degradable network (99.8% DiHDA‐linker + 0.2% Ivocerin). At ambient temperature, the network is rigid and cannot be bend (i). At 100 °C, the network can easily be bended by using tweezers (ii) and cut into pieces (iii). * 1,10‐Decandiol dimethacrylate.

The temperature at which G′ commences to decrease drastically can also be tuned by employing a comonomer, enabling the system to be adapted to different applicational requirements. In the example shown in Figure 2b, isobornyl methacrylate (*i*BoMA) is used as comonomer to form the degradable network and to shift the onset temperature for the decrease in G′ from close to 30 °C for the pure DiHDA‐linker network to close to 50 °C for the copolymer network (*i*BoMA + 0.2 equation DiHDA‐linker). Thus, the system can be fine‐tuned, making it interesting also for applications that require a different debonding temperature.

The drastic decrease in mechanic stability can also be detected by eye as seen in Figure 2c. At ambient temperature, the sample is rigid and cannot be bent or deformed. However, when heated to 100 °C, the sample can easily be bent and cut in half. As the catalyst required for the HDA reaction is removed prior to the network formation, the back reaction to the HDA product and therefore the back formation of the network is disabled, resulting in a permanent cleavage of the network.

To demonstrate the practical applicability of the invented system, adhesion tests were carried out. Therefore, dental crowns were cemented to an implant‐abutment and the pull‐off force was measured at 23 and 80 °C (refer to **Figure** **3**a,b).

**Figure 3:**
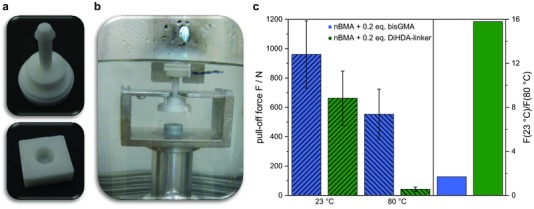
Analysis of the adhesion strength. a) Display of the employed test abutment and crown (for detailed information, refer to the Supporting Information). b) Display of the experimental setup for the pull‐off tests. A water bath was used for temperature control. c) Comparison of the pull‐off forces of a dental model adhesive comonomer system (*n*BMA + 0.2 equation bisGMA*) and a mixture including the designed DiHDA‐linker (*n*BMA + 0.2 equation DiHDA‐linker) at 23 and 80 °C (left part) as well as the calculated differences between the ratios of the pull‐off forces at 23 and 80 °C for both systems (right part). For exact values, refer to the Supporting Information. * Bisphenol‐A‐glycidyl methacrylate.

80 °C can temporarily be employed in the oral cavity, as teeth are known to be poor heat‐conductors.^10^ In the test, the DiHDA‐linker or a dimethacrylate crosslinker (bisGMA), which is commonly used in dental materials, were copolymerized with *n*‐butyl methacrylate (*n*BMA, *T*_g_ of poly(*n*BMA) = 20 °C).^11^ More details regarding the test system and the self‐curing monomer mixture can be found in the Supporting Information. As inspection of Figure 3c indicates, both systems show strong adhesion at 23 °C. However, when heated to 80 °C, only the DiHDA‐linker system shows a drastic decrease in the pull‐off force from 663 N to merely 42 N, resulting in a radical loss of adhesion stability of 94%, compared to only 42% for the common dental adhesion system. When the ratio of the pull‐off force for the two systems at 23 and 80 °C is compared, the drastic difference becomes even clearer (refer to Figure 3c).

To the best of our knowledge, the presented degradable adhesion system is a major improvement over the state of the art, as it is the only adhesive known to combine a high adhesion strength with a fast and easy removability at slightly elevated temperatures.

By designing an adhesive that can be degraded within minutes, we have introduced a polymeric network, which features the properties of a typical adhesive, yet can be destructed on demand. Importantly, the degradation can also be quantified via spectroscopic measurements or even by a simple visual inspection system. By employing different comonomers and/or by using a different HDA‐pair as SRM,^12^ the debonding temperature can be fine‐tuned, making the system interesting for several fields of applications.

## Supporting information

As a service to our authors and readers, this journal provides supporting information supplied by the authors. Such materials are peer reviewed and may be re‐organized for online delivery, but are not copy‐edited or typeset. Technical support issues arising from supporting information (other than missing files) should be addressed to the authors.

Supplementary
