# Supplementary material for: Reversing Adhesion: A Triggered Release Self‐Reporting Adhesive
Source: Adv Sci (Weinh). 2016 Feb 2;3(3):1500361. doi: 10.1002/advs.201500361 (PMC5067662; doi:10.1002/advs.201500361)
Supplement: Supplementary file 1 — Supplementary [file ADVS-3-1500361-s001.pdf]

Copyright WILEY-VCH Verlag GmbH & Co. KGaA, 69469 Weinheim, Germany, 2016.

ADVANCED SCIENCE
Open Access

Supporting Information

for *Adv. Sci.*, DOI: 10.1002/advs.201500361

Reversing Adhesion: A Triggered Release Self-Reporting Adhesive

*Alexander M. Schenzel, Christopher O. Klein, Kai Rist, Norbert Moszner, and Christopher Barner-Kowollik**

DOI: 10.1002/advs.201500361
Article type: Full paper/communication

Supporting Information

Reversing Adhesion – A Triggered Release Self-Reporting Adhesive

*Alexander M. Schenzel, Christopher Klein, Kai Rist, Norbert Moszner and Christopher Barner-Kowollik**

Alexander M. Schenzel, Prof. Christopher Barner-Kowollik

Preparative Macromolecular Chemistry, Institut für Technische Chemie und Polymerchemie, Karlsruhe Institute of Technology (KIT), Engesserstr. 18, DE-76128 Karlsruhe, Germany and Institut für Biologische Grenzflächen, Karlsruhe Institute of Technology (KIT), Hermann-von-Helmholtz-Platz 1, 76344 Eggenstein-Leopoldshafen, Germany.
E-mail: christopher.barner-kowollik@kit.edu

Dr. Christopher Klein

Polymeric Materials, Institut für Technische Chemie und Polymerchemie, Karlsruhe Institute of Technology (KIT), Engesserstr. 18, DE-76128 Karlsruhe, Germany

Prof. Dr. Norbert Moszner, Kai Rist

Ivoclar Vivadent AG
Bendererstr. 2, FL-9494 Schaan, Liechtenstein

Content

1. Materials
2. Characterization
    2.1 Nuclear Magnetic Resonance Spectroscopy
    2.2 Electrospray Ionization-Mass Spectrometry (ESI-MS)
    2.3 UV/Vis Spectroscopy
    2.4 Rheology
    2.5 Pull-off Tests
3. Preparation of the DiHDA-core and -linker
4. Analysis
    4.1 UV/Vis Spectroscopic Measurements
    4.2 Rheological Measurements
    4.3 Pull-off Tests
5. NMR spectra of the prepared substances

1. Materials

1,10-Dibromodecane (97%, Acros), 1-ethyl-3-(3-dimethylaminopropyl)carbodiimide hydrochloride (EDC*HCl, ≥99%, Roth), 2-hydroxyethyl methacrylate (HEMA, 97%, stabilized with MEHQ, Acros), 4-bromomethylbenzoic acid (>95.0%, TCI chemicals), 4-(dimethylamino)pyridine (DMAP, ≥99%, Sigma Aldrich), benzyl methacrylate (BzMA, 96%, stabilized with MEHQ, Sigma Aldrich), n-butyl methacrylate (nBMA, 99%, stabilized with MEHQ, Sigma Aldrich), carbon disulfide (99.9%, VWR), cyclopentadienylsodium (NaCp, 2.0M in THF, Sigma Aldrich), dichloromethane (DCM, 99.8 %, dry, Acros), diethyl phosphite (>95.0%, Fluka), dimethyl sulfoxide (DMSO, ≥99%, Sigma Aldrich), ethyl acetate (EA, 98%, VWR), isobornyl methacrylate (iBoMA, 85-90%, stabilized with MEHQ, Acros), isopropyl methacrylate (iPrMA, >98.0%, stabilized with MEHQ, TCI chemicals), sodium hydride (95%, Sigma Aldrich), tetrahydrofuran (THF, 99.5%, Acros) and zinc chloride (≥98%, Sigma Aldrich) were used as received. 1,10-decanediol dimethacrylate (D_3MA), urethane dimethacrylate (UDMA), bisphenole -A- glycidyl methacrylate (bisGMA) and Ivocerin® were kindly provided by Ivoclar Vivadent AG.

2. Characterization

2.1 Nuclear Magnetic Resonance Spectroscopy (NMR and on-line HT-NMR)

^1H NMR spectroscopy was carried out on a Bruker Acance III 400 spectrometer with a CryoProbe at 400 MHz in $CDCl_3$ with 16 scans for standard measurements and toluene-d_8 in a pressure tube for measurements at elevated temperatures. The temperatures were kept constant by continuous heating with a thermal element (20% heating power), while cooling was performed with a compressed air stream (400 $L \cdot h^{-1}$)

2.2 Electrospray Ionization-Mass Spectrometry (ESI-MS)

Spectra were recorded on an LXQ mass spectrometer (Thermo-Fisher Scientific, San Jose, CA) equipped with an atmospheric pressure ionization source operating in the nebulizer assisted electrospray mode. The instrument was calibrated in the *m/z* range 195 – 1822 using a standard containing caffeine, Met-Arg-Phe-Ala acetate (MRFA) and a mixture of fluorinated phosphazenes (Ultramark 1621) (all from Sigma Aldrich). A constant spray voltage of 6 kV was used and nitrogen at a dimensionless sweep gas flow rate of 2 (approximately 3 L·min^{-1}) and a dimensionless sheath gas flow rate of 5 (approximately 0.5 L·min^{-1}) were applied. The capillary voltage, the tube lens offset voltage and the capillary temperature were set to 10 V, 70 V and 300 °C respectively. The samples were dissolved with a concentration of 0.1 mg·mL^{-1} in a mixture of THF and MeOH (3:2) containing 100 μmol of sodium triflate and infused with a flow of 10 μL·min^{-1}.

2.2 UV-Vis Spectroscopy

UV-visible spectroscopy was performed using a Cary 300 Bio spectrophotometer (Varian) featuring a thermostated sample cell holder. Absorption spectra of the samples were recorded with a resolution of 1 nm and a slit width of 2 nm in a quartz glass cuvette (VWR, quartz glass SUPRASIL®). The absorption spectra of the DiHDA-core were measured in DMSO (ratio: 10 mg mL^{-1}) from 200 nm to 800 nm at temperatures ranging from 25 °C to 140 °C. For the debonding measurements of the networks, the polymerization was performed in the quartz glass cuvettes, ensuring a defined network thickness of 1 mm. The absorption spectra of the networks where recorded from 400 nm to 800 nm at temperatures ranging from 25 °C to 140 °C. From 25 °C to 100 °C the internal heating block auf the UV-Vis spectrophotometer was used to temper the samples. To achieve temperatures above 100 °C an external oil bath

was used. Afterwards, the samples were placed in the UV-Vis spectrophotometer for analysis. As the catalyst required for the HDA reaction is removed prior to the studies, the back reaction to the HDA product is disabled, resulting in a constant absorption.

2.3 Rheology

Instrumental setup

The rheology experimental setup is a TA-Instruments Advanced Rheometric Expansion System Generation 2 (ARES G2), which is a strain controlled rotational rheometer using a separate motor and transducer technology. The motor is an air bearing supported, brushless DC motor, where position and rate are measured with an optical encoder including a position feed-back loop. The rheometer consists of a Force Rebalance Transducer (FRT), which is suitable for measuring torques between 50 nN·m and 200 mN·m as specified by the manufacturer. The installed brushless DC motor with jeweled air bearings is capable of applying rotational angular velocities from 10^{-6} rad/s to 300 rad/s and deformation amplitudes of 1 µrad to an unlimited maximum. The applied oscillation frequency can be varied between 10^{-7} rad/s and 628 rad/s. For temperature control, a force convection oven (FCO) with nitrogen supply was employed.

The temperature dependent measurements were carried out with rectangular specimen (length: 25 mm, width: 5 mm, diameter: 1 mm) employing an axial force of 0.3 N, an excitation frequency of 1 Hz and a deformation of 0.1% in a temperature range of 0 °C to 160 °C (heating rate: 1.5 K/min).

Sample preparation

The networks were prepared via photochemically induced free radical polymerization using 0.2 wt.% Ivocerin® as initiator. The samples were irradiated with 3 Osram Dulux Blue lamps (3 x 18 W, 250 mW/cm^2) for 30 min.

2.4 Pull-off Tests

Preparation of test abutments and crowns

Test abutments (geometry of a truncated cone, diameter top area 4 mm, diameter bottom area 6 mm, height 3 mm) and corresponding test crowns were milled from zirconium oxide. Construction of the crown was done so that placing it on the test abutment resulted in a cement gap of 0.2 mm. Crowns and abutments were sandblasted (110 µm Al_2O_3, 1 bar), cleaned with ultrasonic sound in deionized water for 2 min, dried and prepared for cementation priming with Monobond Plus® (Ivoclar Vivadent AG, handling according to instructions for use: application, wait for 60 s, drying with oil-free air).

Cementation

For cementation, two different self-curing two-component mixtures (mixing ratio 1:1 wt./wt.) with 20 mol% of either the DiHDA-linker (1) or a non-degradable dimethacrylate (bisGMA, 2) were prepared. The first mixture of 1 contained 25.06% nBMA, 0.01% 2,6-di-tert-butyl-p-cresol (BHT), 60.35% DiHDA-linker and 14.58% BP-50-FT (United Initiators). The second mixture of 1 contained 33.33% nBMA, 63.67% DiHDA-linker and 3.00% 3,5-di-tert-butyl-N,N-diethylaniline (DABA). The first mixture of 2 contained 47.90% nBMA, 47.10% bisGMA and 5.00% BP-50-FT (United Initiators). The second mixture of 2 contained 54.30% nBMA, 42.70% bisGMA and 3.00% DABA (declaration in wt.%). The amounts of BP-50-FT and DABA were chosen to obtain a gel time of 1 min to 5 min. 2 drops of a mixture were

thoroughly mixed and applied to the inner face of a primed crown and a primed abutment was put on top. After applying a static load of 2 kg, excess was removed. After 10 min. the weight was removed, and the test specimens were stored at 23 +/- 2 °C for 3 days. Overall 10 test specimens were prepared of each mixture.

Pull-oft tests

For the determination of the pull-off forces, the test specimens of each mixture were randomly divided into 2 groups and mounted to a tensile testing machine (Zwick-Roell Z010). The test specimens of the first group were tempered in a water bath at 23 °C for 1 min. prior to the measurement. For the second group, the water bath was heated to 80 °C. The crowns were pulled-off the abutments in the water bath, with a constant crosshead speed of 1.0 mm/min and the maximum force was determined.

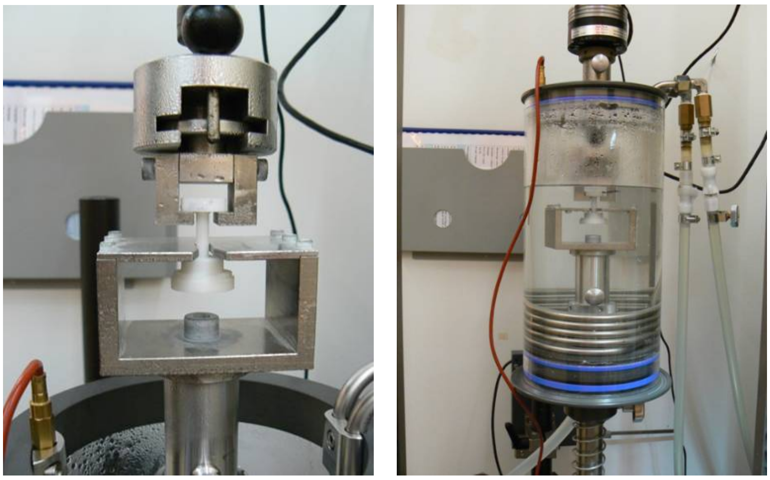

Figure S1. Display of the employed tensile testing machine (Zwick-Roell Z010). The pull-off tests were carried out in a heatable water bath in order to ensure the temperature control.

3. Preparation of the DiHDA-core and linker

3.1 Synthesis of 4-((((Diethoxyphosphoryl)carbonothioyl)thio)methyl)-benzoic acid (PDTMBA)

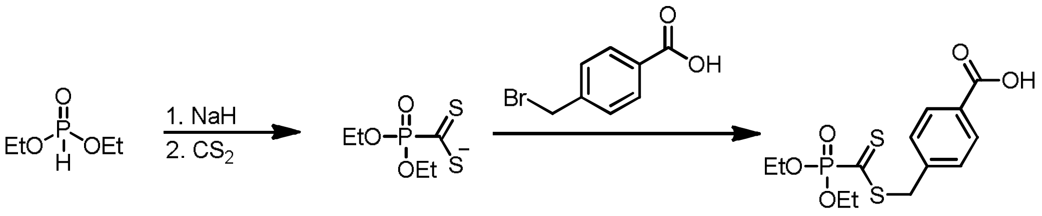

Scheme S1. Preparation of PDTMBA.

A solution of 5.3 mL diethyl phosphite (1.0 eq. 41 mmol, 5.7 g) in 40 mL dry THF is added to a stirred suspension of 1.5 g NaH (1.5 eq., 62 mmol) in 20 mL dry THF at ambient temperature. After completion of the H_2 formation, the reaction mixture is heated to reflux for 15 min. Subsequently, the solution is cooled to -90 °C and 12.3 mL CS_2 (5.0 eq., 205 mmol, 15.6 g) are added dropwise. It is stirred for 2 h, whereat the reaction mixture turns brown. After the addition of 750 mL THF to facilitate the stirring, a solution of 10.0 g 4-bromomethylbenzoic acid (1.1 eq., 46 mmol) in 75 ml THF is added dropwise at ambient temperature. After stirring for 16 h, the solvent is removed from the purple reaction mixture. DCM and H_2O are added in a ratio of 1:1 until all residue is dissolved. The water phase is washed with DCM and the combined organic layers are dried over Na_2SO_4. After removal of the solvent, the crude product is purified via column chromatography (cyclohexane : EA : acetic acid = 1:1:0.01) to give a purple solid of **PDTMBA** (4.3 g, 30% yield).

^{1}H NMR (400 MHz, $CDCl_3$, δ, ppm): 1.37 (t, 6H, $OCH_2C\mathbf{H}_3$), 4.22 - 4.34 (m, 4H, $OC\mathbf{H}_2CH_3$), 4.54 (s, 2H, SCH_2), 7.41 (d, 2H, ArH), 8.05 (d, 2H, ArH), 10.50 (bs, 1H, COOH). **^{13}C NMR** (101 MHz, $CDCl_3$, δ, ppm): 16.40 ($OCH_2\mathbf{C}H_3$), 40.00 (SCH_2), 65.14 ($O\mathbf{C}H_2CH_3$), 129.34 (C_{ar}), 129.53 (C_{ar}), 130.79 (C_{ar}), 140.04 (C_{ar}), 170.73 (COOH), 226.92 (PC=S), 228.66 (PC=S).

3.2 Synthesis of 1,10-dicyclopentadienyl decane (1,10-DiCp-decane)

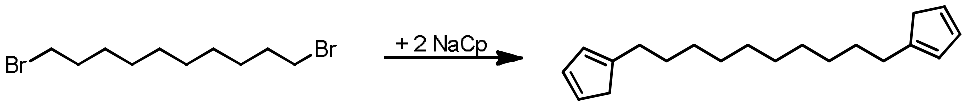

Scheme S2. Preparation of 1,10-DiCp-decane.

To a cooled solution of 6.0 g 1,10-dibromodecane (1.0 eq., 20 mmol) in 30 mL dry THF 20 mL of a 2M solution of NaCp in THF (2.0 eq., 40 mmol) are added at -5 °C. After stirring for 1 h at -5 °C, the reaction mixture is allowed to warm to ambient temperature and is stirred overnight. Subsequently, the suspension is filtered over silica gel with 300 mL EA. The solvent is removed and the residue is taken up with n-hexane. The solution is again filtered of silica gel with 1.5 L n-hexane. After removal of the solvent, a clear and viscous liquid of **1,10-DiCp-decane** was obtained (4.7 g, 87% yield).

^{1}H NMR (400 MHz, $CDCl_3$, *δ*, ppm): 1.29 - 1.45 (m, 12H, $(C\mathbf{H}_2)_3CH_2CH_2Cp$), 1.50 - 1.65 (m, 4H, $(CH_2)_3C\mathbf{H}_2CH_2Cp$), 2.40 - 2.50 (m, 4H, $(CH_2)_3CH_2C\mathbf{H}_2Cp$), 2.97 (d, 4H, $CH_{2,Cp}$), 6.05 - 6.53 (m, 6H, CpH). **^{13}C NMR** (101 MHz, $CDCl_3$, *δ*, ppm): 29.02 (CH_2), 29.62 (CH_2), 29.68 (CH_2), 29.77 (CH_2), 29.91 (CH_2), 30.01 (CH_2), 30.87 ($CH_2\mathbf{C}H_2Cp$), 41.27 (C_{Cp}), 43.42 (C_{Cp}), 125.72 (C_{Cp}), 126.26 (C_{Cp}), 130.36 (C_{Cp}), 132.56 (C_{Cp}), 133.58 (C_{Cp}), 134.95 (C_{Cp}), 147.49 ($CH_2CH_2\mathbf{C}_{Cp}$), 150.21 ($CH_2CH_2\mathbf{C}_{Cp}$).

3.3 Synthesis of 2-(methacryloyloxy)ethyl 4-((((diethoxyphosphoryl)carbonothioyl)thio)methyl)benzoat (MA-PDTMBA))

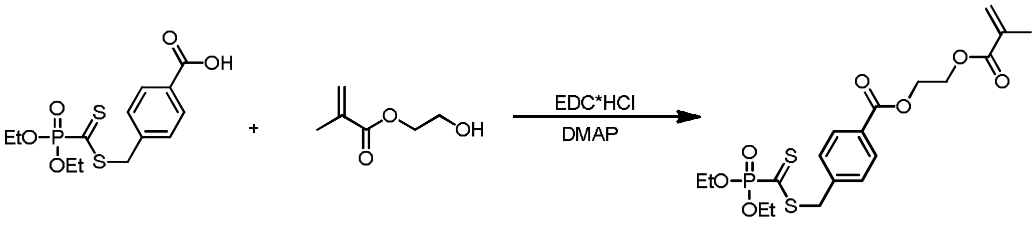

Scheme S3. Preparation of MA-PDTMBA.

To a solution of 2.0 g PDTMBA (1.0 eq., 5.74 mmol) and 1.05 mL HEMA (1.5 eq., 8.61 mmol, 1.12 g) in dry DCM, a solution of 2.2 g ECD*HCl (2.0 eq., 11.5 mmol) and 0.14 g DMAP (0.2 eq., 1.15 mmol) in dry DCM is added at ambient temperature. After stirring for 20 h, the solution is washed with $NaHCO_3$ solution and brine. The organic layer is dried over Na_2SO_4 and the solvent is removed. The crude product is purified via column chromatography (cyclohecane : EA = 1:1) to yield a purple liquid of **MA-PDTMBA** (1.85 g, 70% yield).

^{1}H NMR (400 MHz, $CDCl_3$, δ, ppm): 1.37 (t, 6H, $OCH_2C\mathbf{H}_3$), 1.90 (s, 3H, CH_3C_{MA}), 4.22 - 4.34 (m, 4H, $OC\mathbf{H}_2CH_3$), 4.42 - 4.58 (m, 4H, $OC\mathbf{H}_2C\mathbf{H}_2O$), 4.52 (s, 2H, SCH_2), 5.55 (d, 1H, $CH_{2,trans}$), 6.10 (d, 1H, $CH_{2,cis}$), 7.37 (d, 2H, ArH), 7.98 (d, 2H, ArH). **^{13}C NMR** (101 MHz, $CDCl_3$, δ, ppm): 16.40 ($OCH_2\mathbf{C}H_3$), 18.40 ($C_{MA}\mathbf{C}H_3$), 40.00 (SCH_2), 62.60 ($O\mathbf{C}H_2\mathbf{C}H_2O$), 65.14 ($O\mathbf{C}H_2CH_3$), 126.27 ($\mathbf{C}H_2C_{MA}$), 129.48 ($C_{ar}$), 129.65 ($C_{ar}$), 130.28 ($C_{ar}$), 136.03 ($C_{MA}$), 139.50 ($C_{ar}$), 165.90 ($C_{ar}\mathbf{C}$=O), 167.26 ($C_{MA}\mathbf{C}$=O), 227.18 (PC=S), 228.93 (PC=S).

3.4 Synthesis of the DiHDA-core

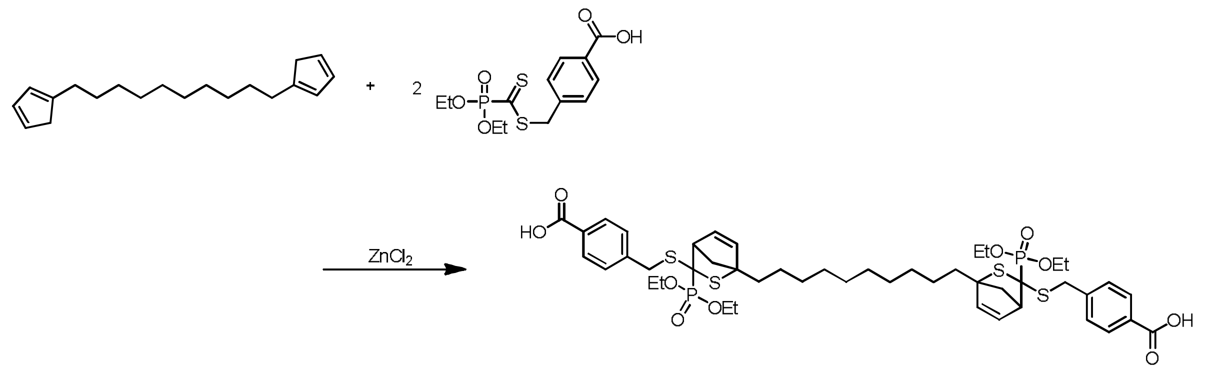

Scheme S4. Preparation of the DiHDA-core.

3.0 g PDTMBA (2.00 eq, 8.61 mmol), 1.16 g 1,10-DiCp-decane (1.00 eq., 4.31 mmol) and 63 mg $ZnCl_2$ (0.10 eq., 0.46 mmol) are dissolved in EA and stirred at ambient temperature for

30 min. Subsequently, the organic layer is washed with water. After removal of the solvent a white solid of the **DiHDA-core** is obtained (4.13 g, 99% yield).

^{1}H NMR (400 MHz, $CDCl_3$, *δ*, ppm): 1.25 - 1.65 (m, 28H, $OCH_2C\mathbf{H}_3$, $C_{HDA}CH_2(C\mathbf{H}_2)_4$), 1.95 - 2.18 (m, 4H, $CH_{2,HDA\text{-}Brücke}$), 2.18 - 2.50 (m, 4H, $C_{HDA}C\mathbf{H}_2(CH_2)_4$), 3.35 - 3.68 (m, 2H, CH_{HDA}), 4.00 - 4.25 (m, 8H, $OC\mathbf{H}_2CH_3$), 4.26 - 4.44 (m, 4H, SCH_2C_{ar}), 5.52 - 5.60 (m, 1H, $CH_{HDA\text{-}Db.}$), 5.95 - 6.03 (2H, $CH_{HDA\text{-}Db.}$), 6.28 - 6.33 (m, 1H, $CH_{HDA\text{-}Db.}$), 7.42 (d, 4H, ArH), 7.95 (d, 4H, ArH), 10.10 (bs, 2H, COOH). **ESI-MS**: *m/z* calculated 966.29, found 966.29.

3.5 Synthesis of the DiHDA-linker

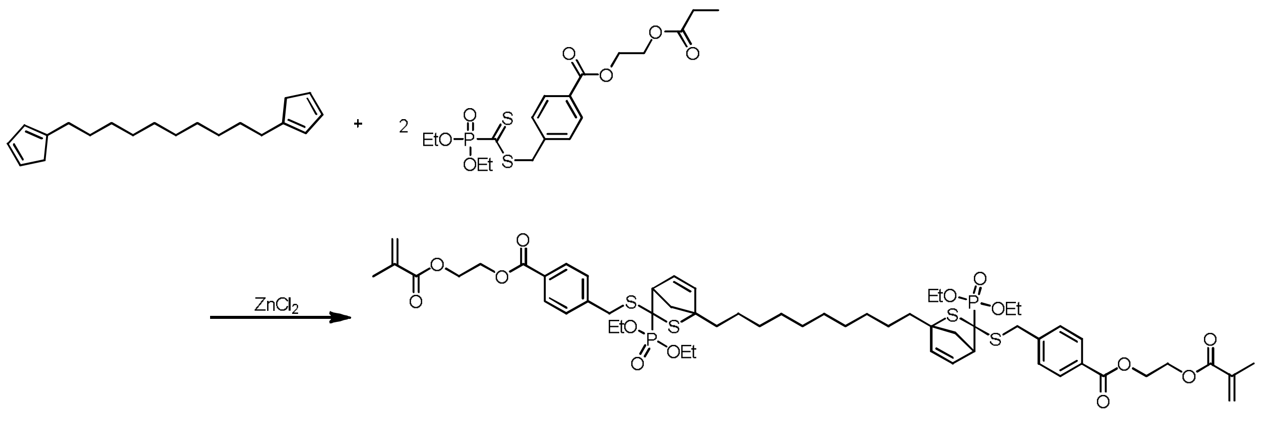

Scheme S 5. Preparation of the DiHDA-linker.

0.59 g 1,10-DiCp-decane (1.0 eq., 2.17 mmol), 2.0 g MA-PDTMBA (2.0 eq., 4.34 mmol) and 30 mg $ZnCl_2$ (0.1 eq., 0.22 mmol) are dissolved in EA and stirred at ambient temperature for 30 min. Subsequently, the organic layer is washed with water. After removal of the solvent, a highly viscous liquid is obtained (2.56 g, 99% yield).

^{1}H NMR (400 MHz, $CDCl_3$, *δ*, ppm): 1.25 - 1.65 (m, 28H, $OCH_2C\mathbf{H}_3$, $C_{HDA}CH_2(C\mathbf{H}_2)_4$), 1.94 (s, 6H, CH_3C_{MA}), 1.95 - 2.18 (m, 4H, $CH_{2,HDA\text{-}Brücke}$), 2.18 - 2.50 (m, 4H, $C_{HDA}C\mathbf{H}_2(CH_2)_4$), 3.35 - 3.68 (m, 2H, CH_{HDA}), 4.00 - 4.25 (m, 8H, $OC\mathbf{H}_2CH_3$), 4.26 - 4.44 (m, 4H, SCH_2C_{ar}), 4.45 – 4.58 (m, 8H, $OC\mathbf{H}_2C\mathbf{H}_2O$), 5.52 - 5.60 (m, 1H, $CH_{HDA\text{-}Db.}$), 5.58 (d, 2H, $CCH_{2,trans}$),

5.95 - 6.03 (2H, $CH_{HDA\text{-}Db.}$), 6.13 (d, 2H, $CCH_{2,cis}$), 6.28 - 6.33 (m, 1H, $CH_{HDA\text{-}Db.}$), 7.42 (d, 4H, ArH), 7.95 (d, 4H, ArH). **ESI-MS**: *m/*z calculated 1190.39, found 1190.37.

4. Analysis

4.1 UV/Vis Spectroscopic Measurements

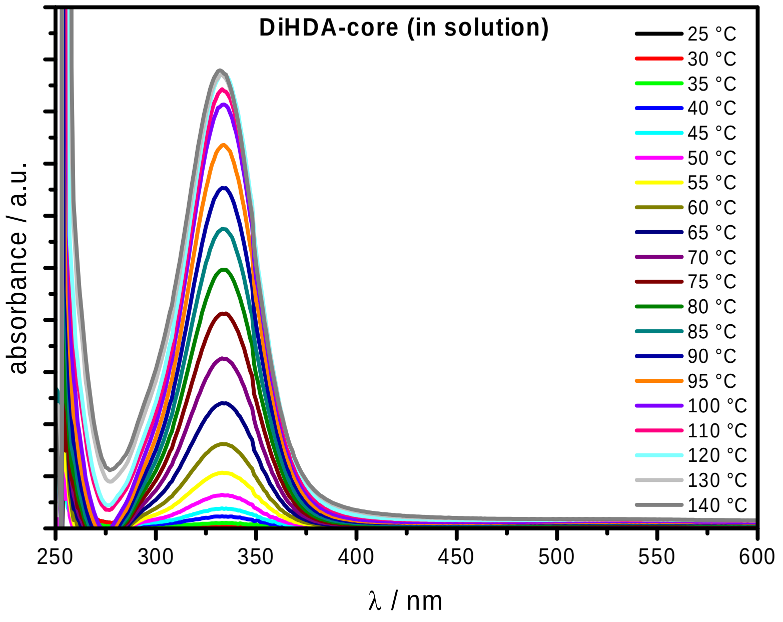

Figure S2. UV/Vis spectroscopy of the DiHDA-core (in solution).

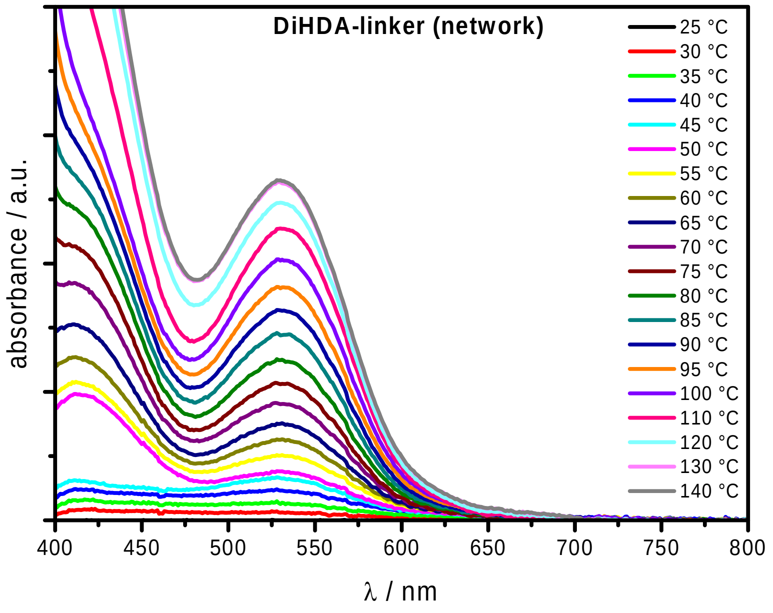

Figure S3. UV/Vis spectroscopy of the DiHDA-linker (network).

4.2 Rheological Measurements

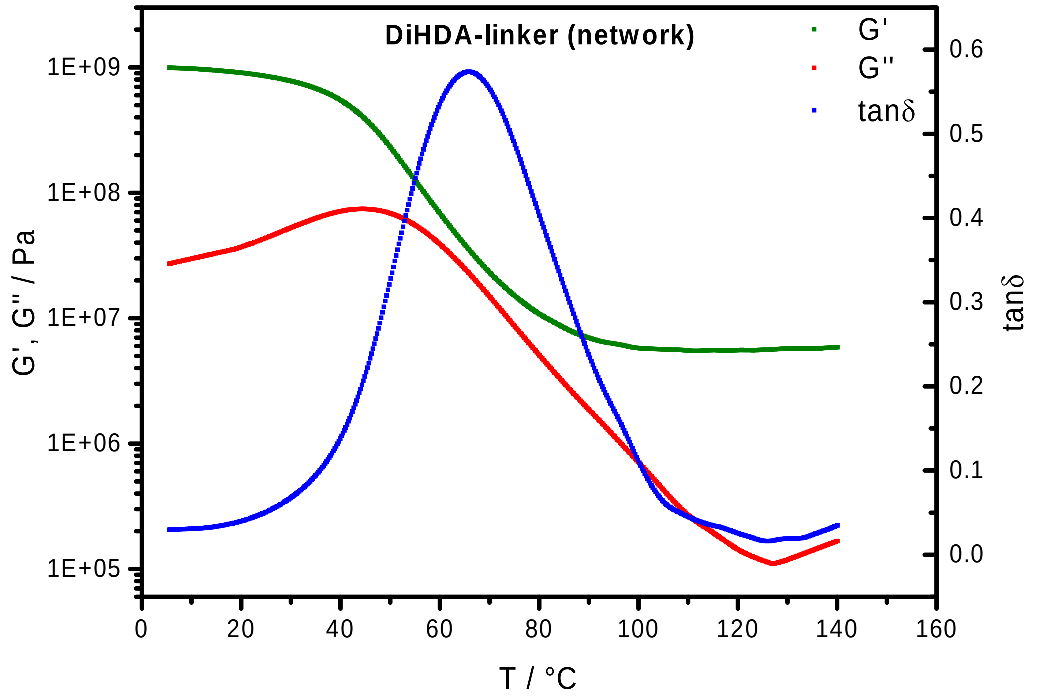

Figure S4. Rheological measurement of a network based on pure DiHDA-linker (preparation see 2.3 Rheology – sample preparation). Displayed are the storage modulus (G'), the loss modulus (G'') and tanδ (G''/G').

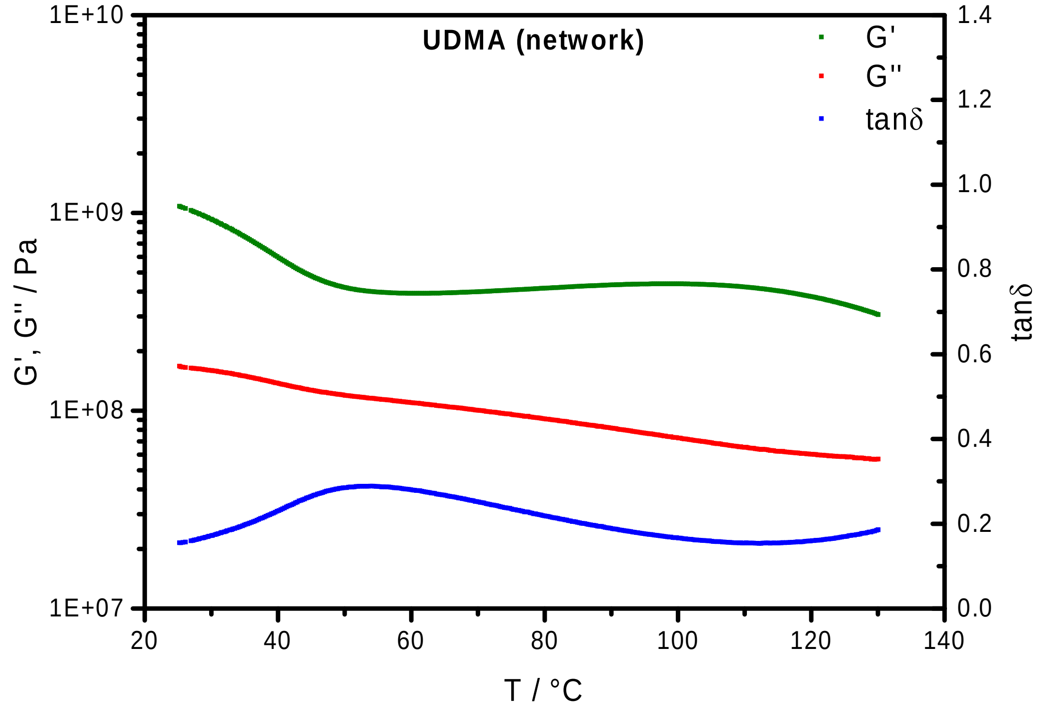

Figure S5. Rheological measurement of a network of pure UDMA (non-degradable dimethacrylate, preparation see 2.3 Rheology – sample preparation). Displayed are G', G'' and tanδ.

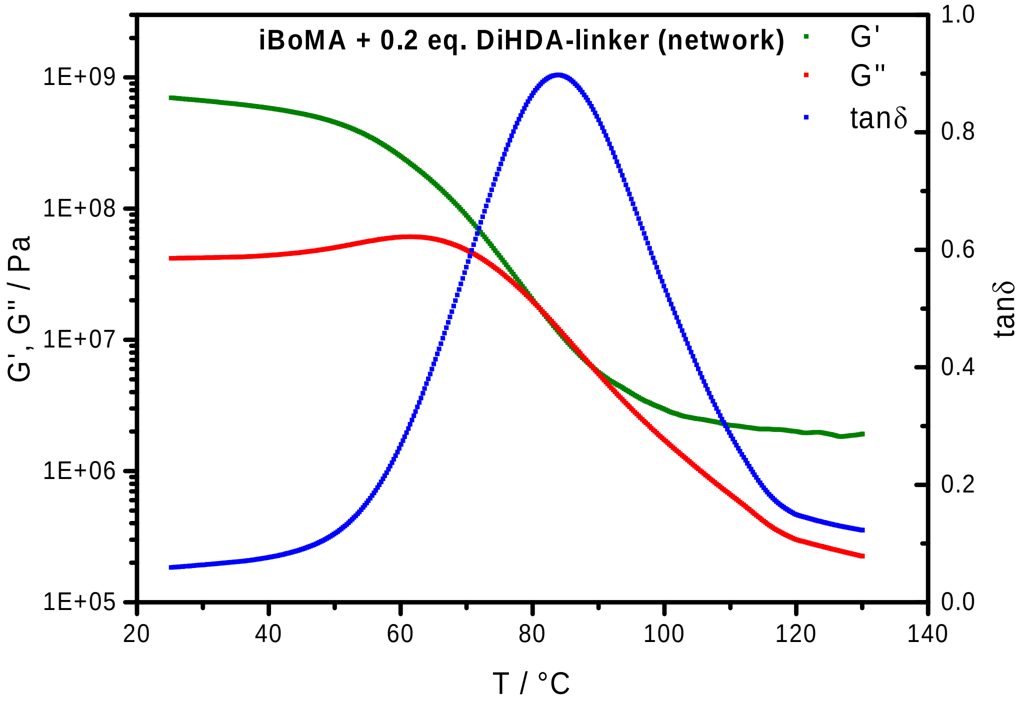

Figure S6. Rheological measurement of a network of iBoMA and 0.2 eq. (20 mol%) of the DiHDA-linker (preparation see 2.3 Rheology – sample preparation). Displayed are G', G'' and tanδ.

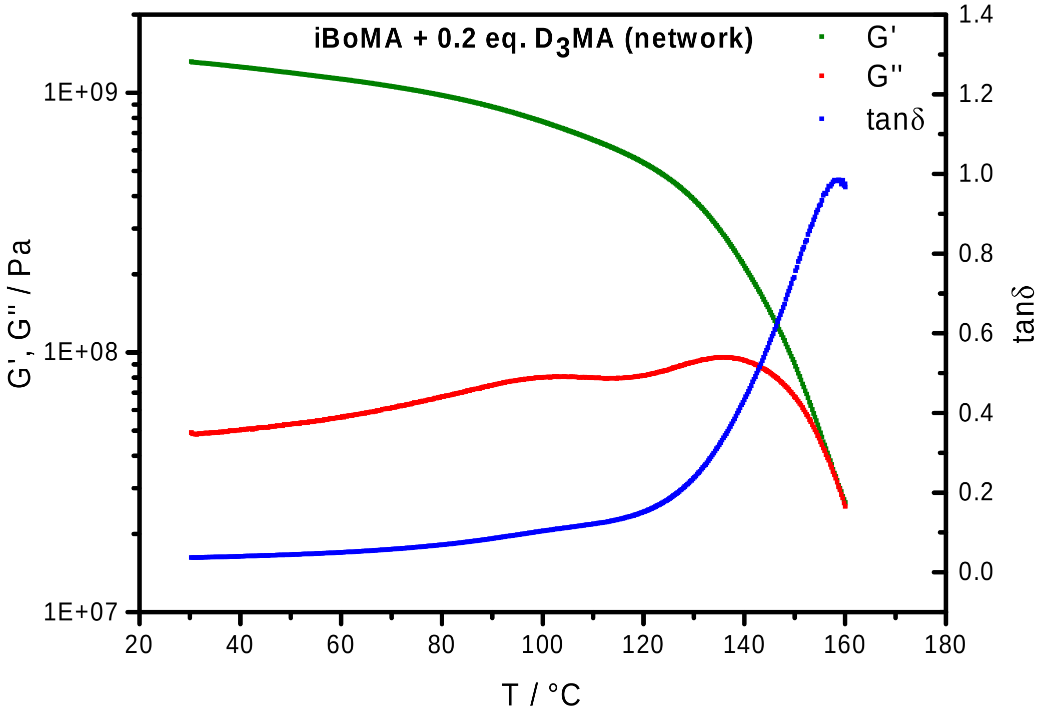

Figure S7. Rheological measurement of a network of isobornyl methacrylate (iBoMA) and 0.2 eq. (20 mol%) of a non-degradable dimethacrylate (D_3MA, preparation see 2.3 Rheology – sample preparation). Displayed are G', G'' and tanδ.

4.3 Pull-off Tests

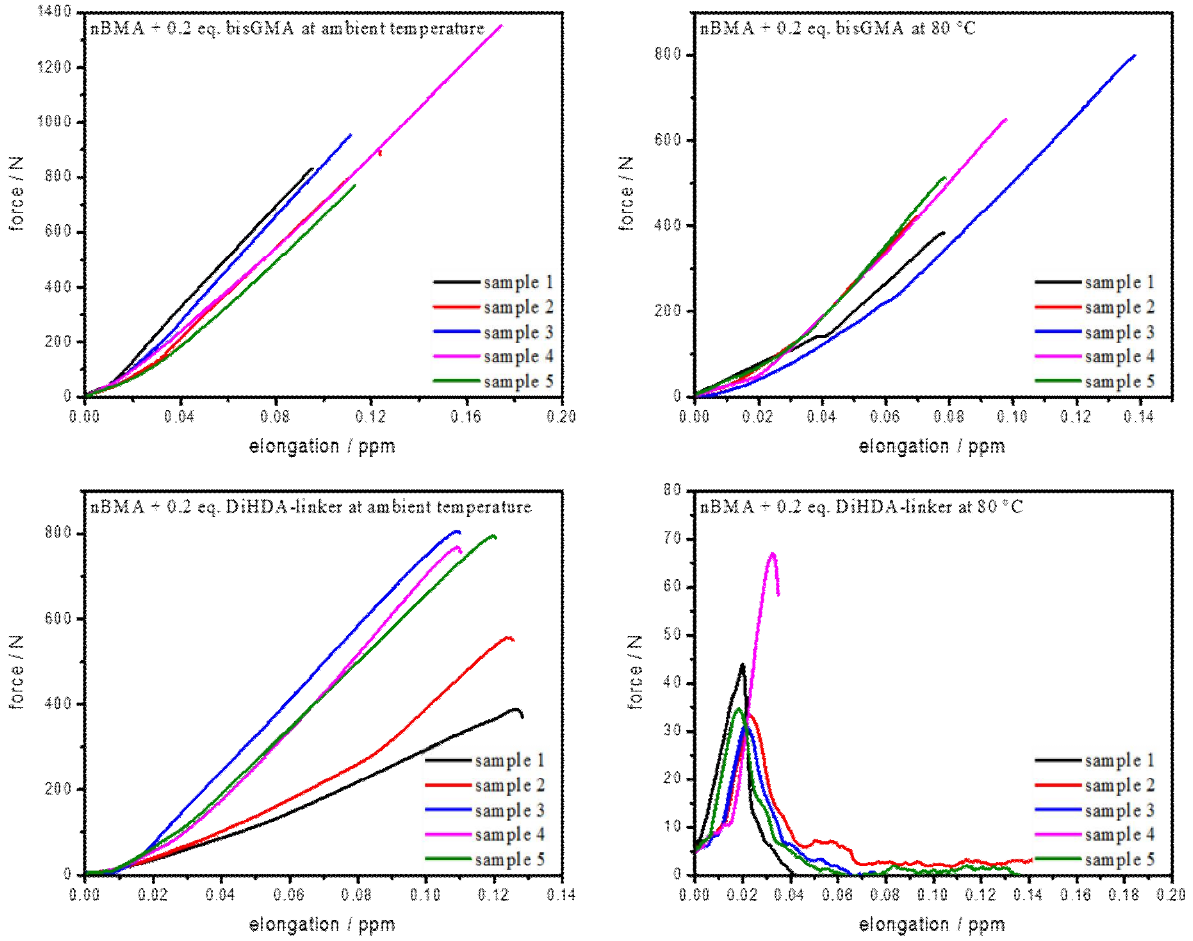

Figure S7. Force-elongation graphs of the performed pull-off tests of test specimens bound with two different self-curing two-component mixtures entailing nBMA and 0.2 eq. of either a non-degradable dimethacrylate (bisGMA) or the DiHDA-linker at ambient temperature and at 80 °C.

	**nBMA + 0.2 eq. bisGMA***	**nBMA + 0.2 eq. DiHDA-linker***
**F (23 °C)**	962 N (+/- 229 N)	663 N (+/- 184 N)
**F (80 °C)**	553 N (+/- 171 N)	42 N (+/- 15 N)

Table S1. Values determined for the pull-off force F of test specimens bound with two different self-curing two-component mixtures with nBMA and 0.2 eq. of either a non-degradable dimethacrylate (bisGMA) or the DiHDA-linker. Detailed information regarding the preparation of the test specimens are provided in section 2.4 Pull-off Tests. *BP-50-FT or DABA as Initiator.

5. NMR spectra of the prepared substances

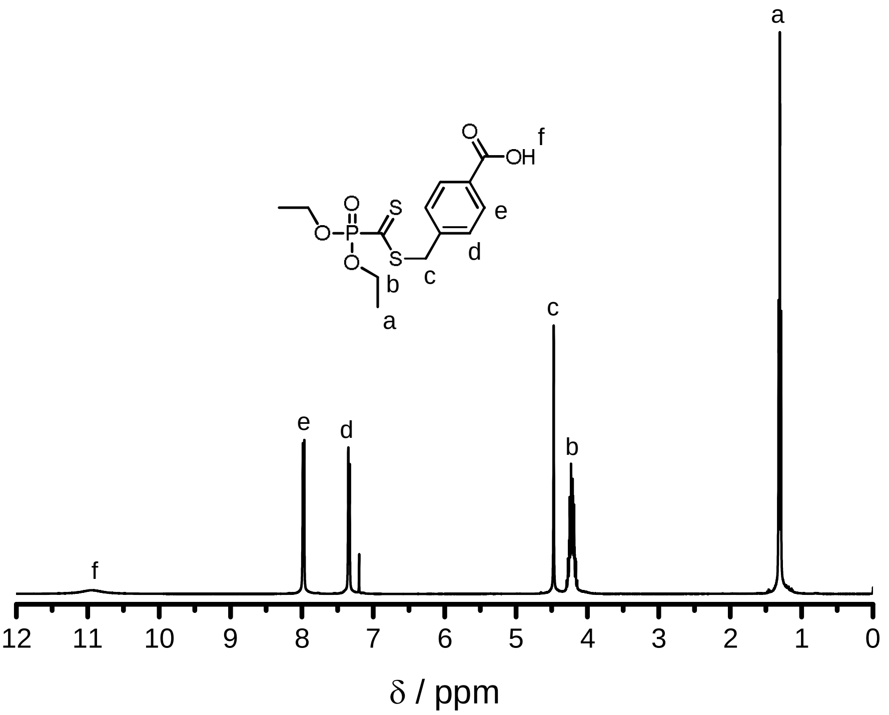

Figure S8. ^{1}H NMR spectrum of PDTMBA in $CDCl_3$ at ambient temperature.

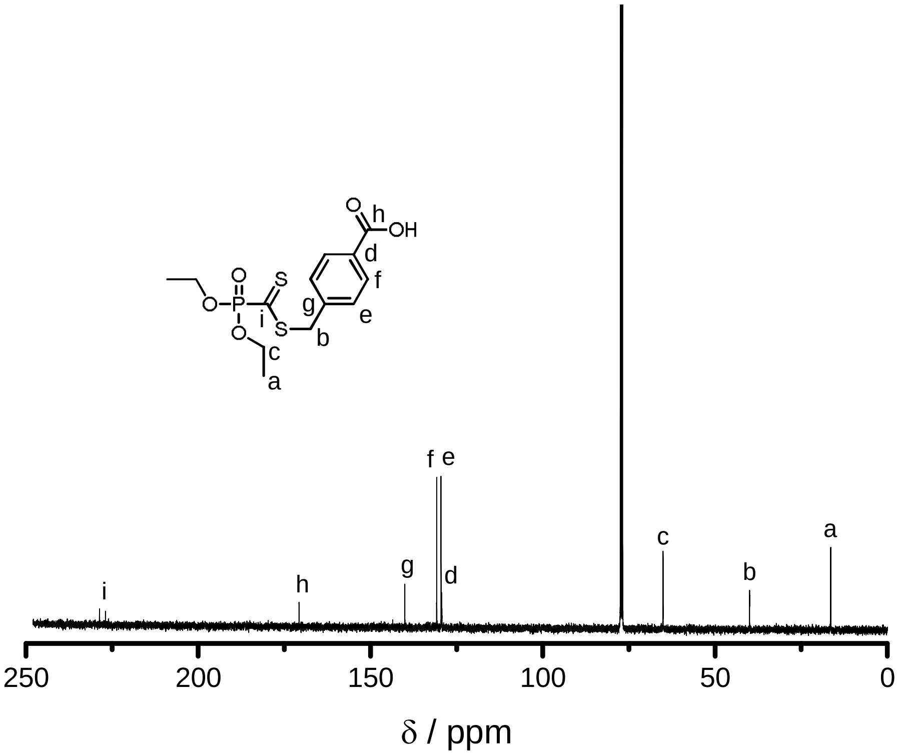

Figure S9. ^{13}C NMR spectrum of PDTMBA in $CDCl_3$ at ambient temperature.

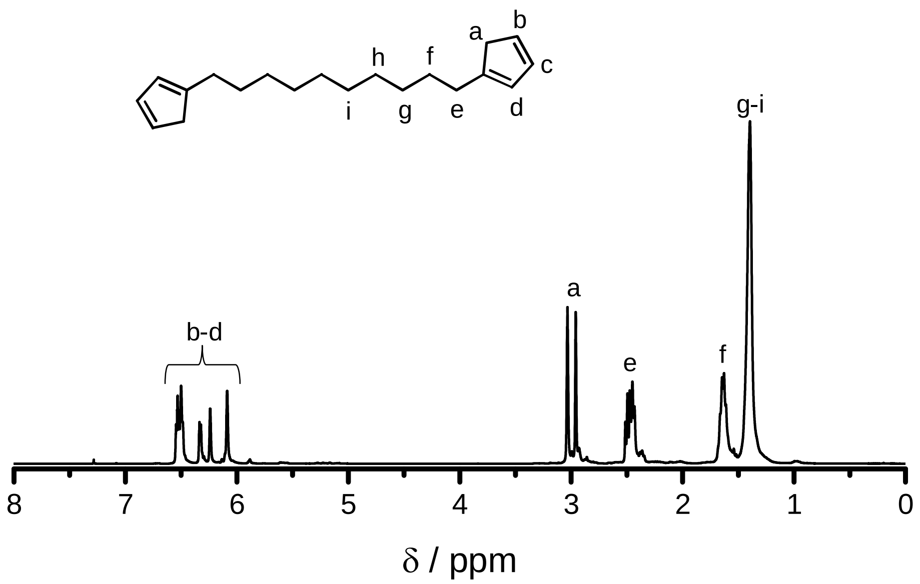

Figure S10. ^{1}H NMR spectrum of 1,10-DiCp-decane in $CDCl_3$ at ambient temperature.

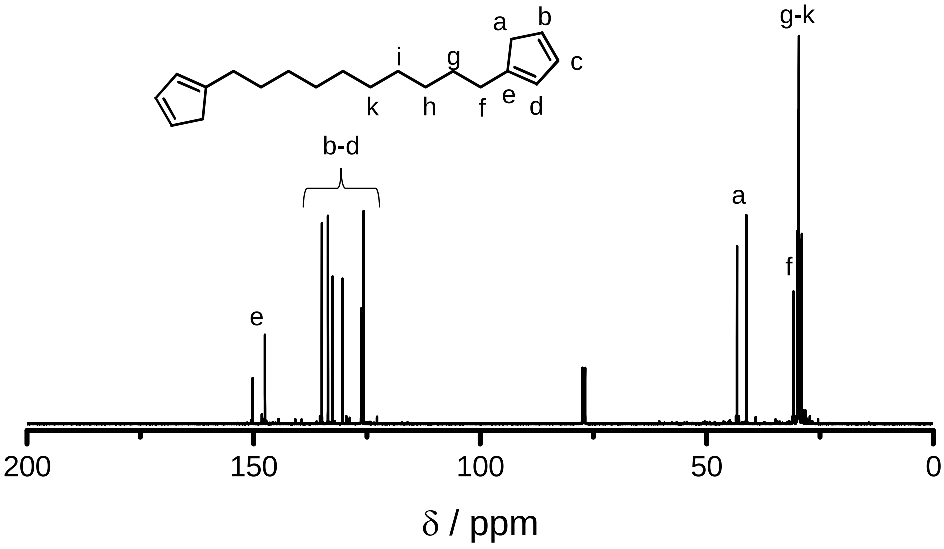

Figure S11. ^{13}C NMR spectrum of 1,10-DiCp-decane in $CDCl_3$ at ambient temperature.

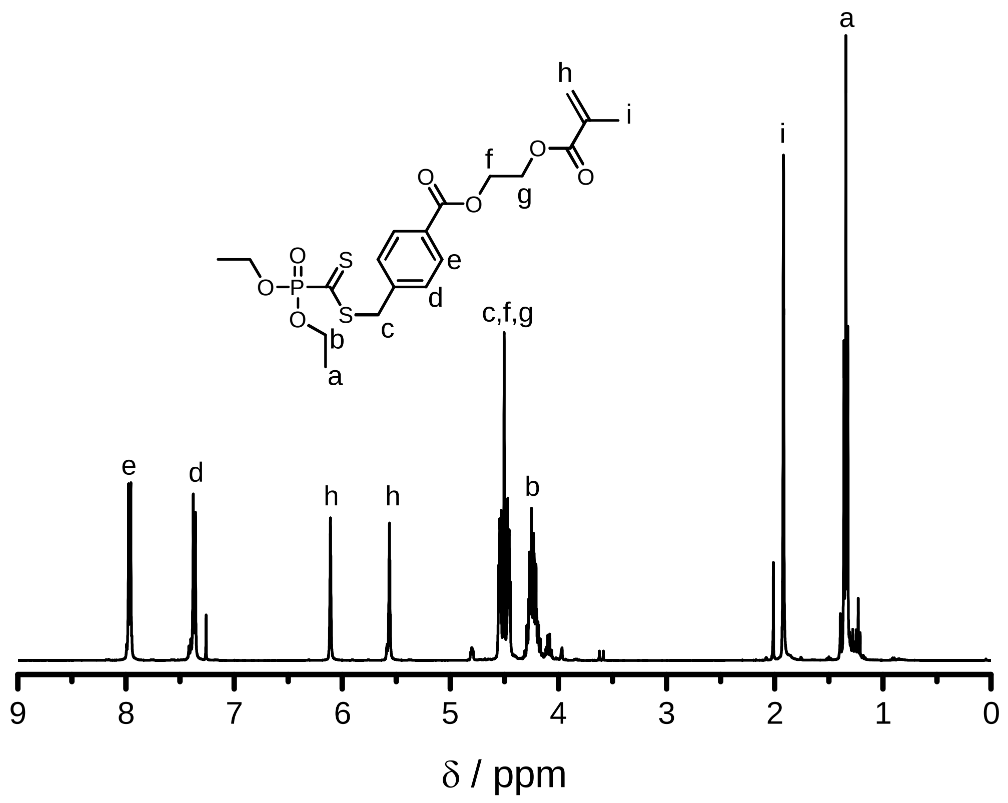

Figure S12. ^{1}H NMR spectrum of MA-PDTMBA in $CDCl_3$ at ambient temperature.

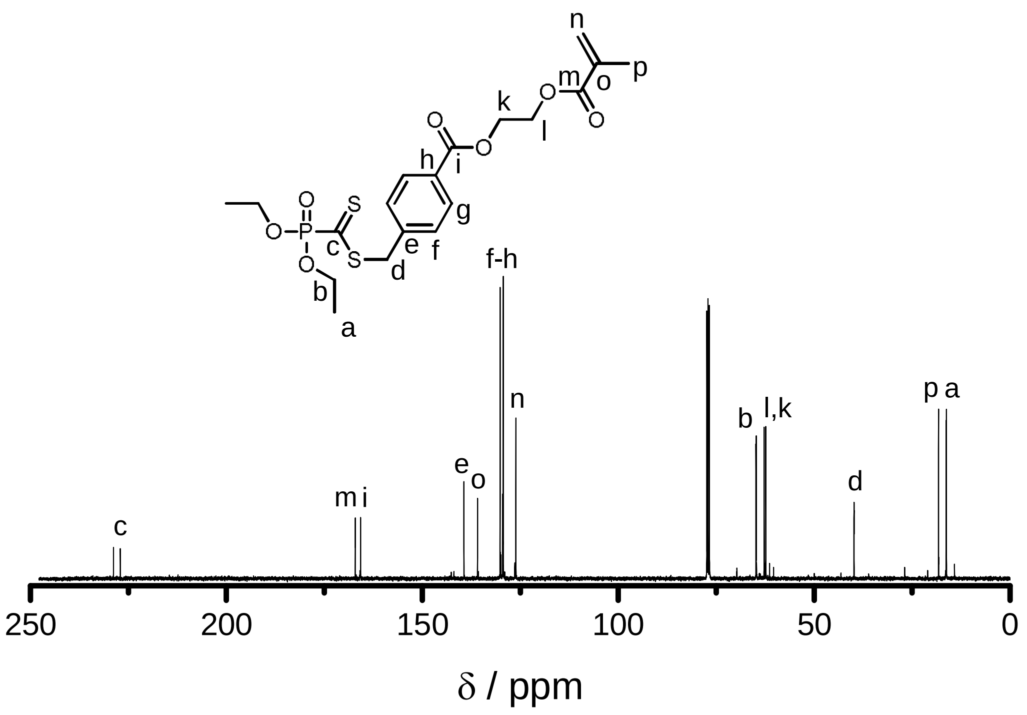

Figure S13. ^{13}C NMR spectrum of MA-PDTMBA in $CDCl_3$ at ambient temperature.

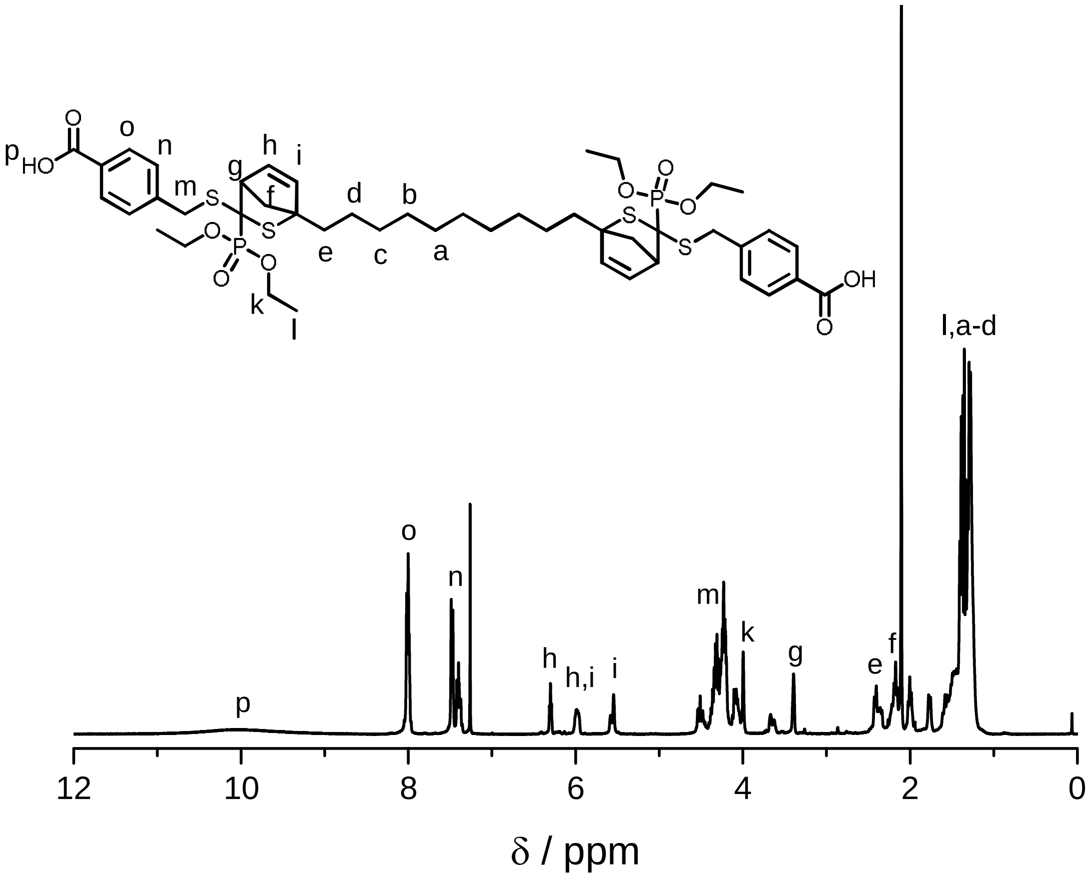

Figure S14. ^{1}H NMR spectrum of the DiHDA-core in $CDCl_3$ at ambient temperature.

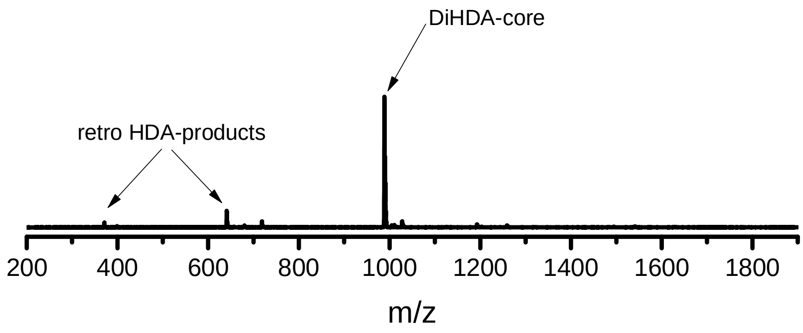

Figure S15. ESI mass spectrum of the DiHDA-core. The retro HDA products are formed during the ionization process due to the high temperatures (320 °C).

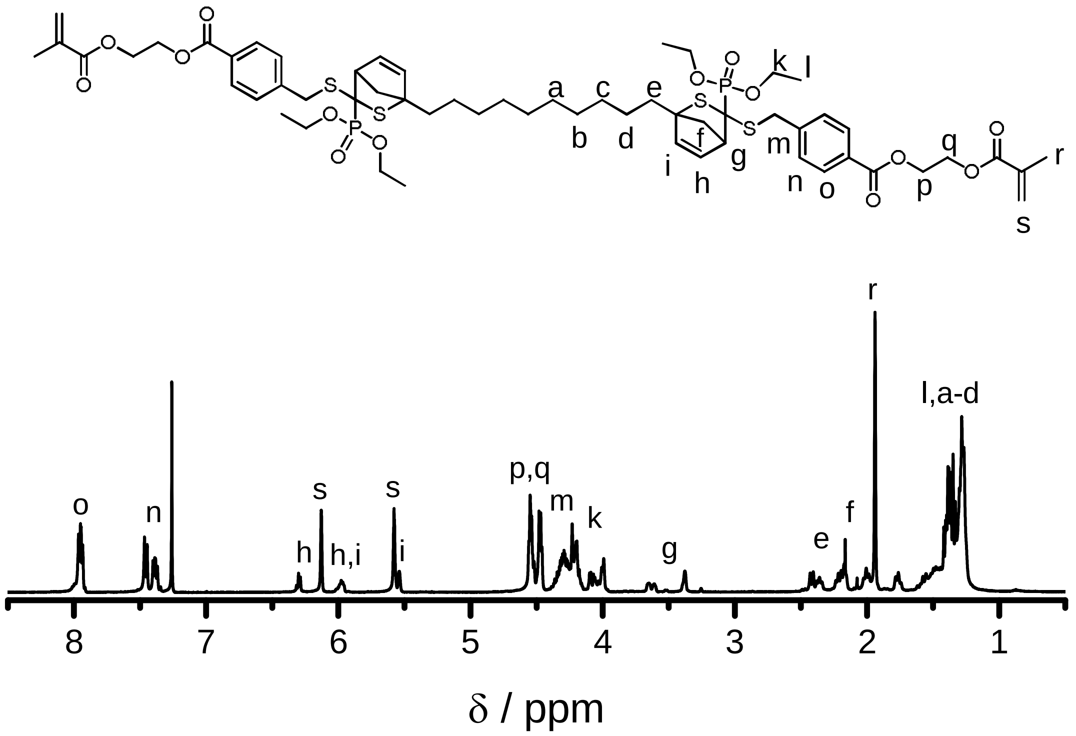

Figure S16. ^{1}H NMR spectrum of the DiHDA-linker in $CDCl_3$ at ambient temperature.

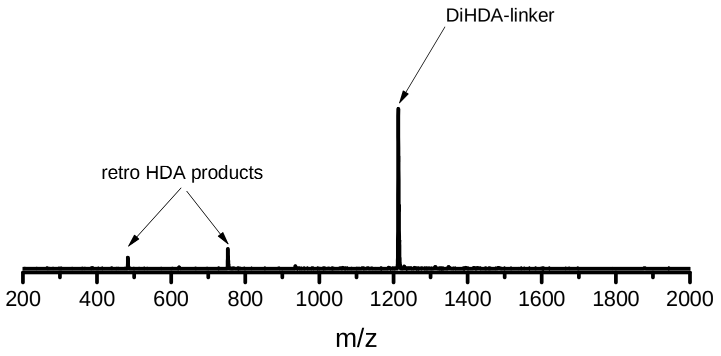

Figure S17. ESI mass spectrum of the DiHDA-linker. The retro HDA products are formed during the ionization process due to the high temperatures (320 °C).

Copyright WILEY-VCH Verlag GmbH & Co. KGaA, 69469 Weinheim, Germany, 2013.
